# Difficulties Associated With Spiritual Care According to the Nursing Staff at an Oncology Clinic: A 20‐Year Longitudinal Mixed Method Case Study

**DOI:** 10.1111/jan.16929

**Published:** 2025-03-26

**Authors:** Mikael Lundmark

**Affiliations:** ^1^ Department of Historical, Philosophical and Religious Studies Umeå University Umeå Sweden

**Keywords:** caregiving, case study research, holistic care, spirituality

## Abstract

**Aim(s):**

To replicate a study conducted twenty years ago regarding difficulties associated with spiritual care at an oncology clinic. Compare the results against societal changes, educational changes and changes at the clinic.

**Design:**

Mixed‐method, longitudinal, descriptive and comparative design.

**Methods:**

A questionnaire‐based replication‐study conducted in 2003 and 2023 with comparative and categorical content analyses. The questionnaire was handed out to all nursing staff at duty at the investigate oncology clinic during the data collection periods.

**Results:**

The difficulties associated with spiritual care in 2003 and 2023 are very similar and include (i) a lack of knowledge and education; (ii) the nurses’ own approaches to spiritual care can be perceived as having a negative impact, and (iii) practical or structural difficulties, including a lack of time, experiences of stress, a lack of suitable locations for individual conversations.

**Conclusion:**

The difficulties identified in the 2023 study mirror previous research to a high degree. These difficulties have not, in any substantial way, changed between 2003 and 2023. This conclusion indicates that difficulties associated with spiritual care might not be sensitive to societal changes regarding spirituallity, organisational changes at the clinic, or educational changes regarding the nurses’ formal education in spirituality and healthcare.

**Implications for the Profession and/or Patient Care:**

This study is informative with regard to identifying actions that can obviate difficulties associated with spiritual care.

**Reporting Method:**

When applicable: SRQR.

**Patient or Public Contribution:**

None.

## 1 Introduction

This longitudinal study investigates perceived difficulties associated with spiritual care at an oncology clinic to provide insight into whether such difficulties are sensitive to societal changes related to spirituality, organisational changes at the clinic or changes in the nurses' formal education regarding spirituality and health care. This investigation also provides valuable insights, which ought to be considered when implementing or evaluating studies on spiritual care in other healthcare contexts and in other parts of the world.

Since the end of the 20th century, awareness of patients' spiritual needs has increased substantially in the healthcare context (O'Brien 2022). Today, the connection between spirituality, at least when it relates to religion, and health is well‐established in numerous studies (Koenig et al. 2024). The significance of this awareness has been studied and discussed in various nursing contexts including, but not limited to, general practice nursing (Schreiber et al. 2022), critical care (García Torrejon et al. 2023), mental health nursing (Elliott et al. 2020), nursing homecare (Santos et al. 2021) and, perhaps especially, within cancer care (Van Meurs et al. 2018) and palliative care (de Brito Sena et al. 2021). Lundberg et al. (2024) show in a survey of 18 original articles within palliative care research that it is assumed that every individual has a spiritual dimension, and healthcare professionals are expected to be able to provide spiritual care. Recurring topics in research on spirituality and health care include questions about what determines spiritual care practices (Mascio et al. 2022), competencies for spiritual care (Espinel et al. 2022), how to understand the concepts of ‘spirituality’ (de Brito Sena et al. 2021) and ‘spiritual care’ (Hvidt et al. 2020) and difficulties/barriers associated with the provision of spiritual care (Balboni et al. 2014; Jones et al. 2021; Mascio et al. 2022; Van Meurs et al. 2018).

Several conceptual and methodological problems associated with research on spirituality and health have also been identified (Reinert and Koenig 2013; Chagas et al. 2023). For example, concerns have been raised about the impact of cultural differences on the manner in which the concepts of ‘spirituality’ and ‘spiritual care’ are understood in different healthcare contexts (Torskenæs et al. 2015; Taylor et al. 2023). Similarly, questions concerning the challenges associated with providing spiritual care have been investigated (Garssen et al. 2017). However, one question that has not been addressed, as far as I have been able to find, is whether difficulties associated with providing spiritual care remain stable over time or whether they are sensitive to, for example, societal changes in general, changes in the nurses' formal education regarding spirituality and health care, or organisational changes at the workplace.

To investigate this question, a study was conducted in 2023 that replicated a 2003 study on the understanding of, attitudes towards and difficulties associated with spiritual care among nursing staff in a holistic nursing context represented by an oncological clinic in Sweden (Lundmark 2003, 2005, 2006). In this study, a *holistic nursing context* refers to a context of nursing that responds to an individual's physical, psychological, social and spiritual needs (Margereson and Trenoweth 2009). Different problems associated with spiritual care were identified in the 2003 study and thematised into six major themes, reproduced in Table 1. Given the nature of the difficulties identified in 2003, one may argue that they are a product of organisational, educational, individual or societal issues. Therefore, when examining what the situation regarding perceived difficulties associated with spiritual care looked like in 2023, it is of interest to know what has happened since 2003 on the societal level in general (including societal attitudes towards spirituality, presumably mirrored by the nursing staff's attitudes at the studied clinic), and regarding organisational changes at the clinic studied. In addition, possible changes in the nurses' formal education regarding questions about spirituality and health are also of relevance.

**TABLE 1:** Categories, subcategories, and sub‐subcategories generated from a content analysis of the answers to the question: “What difficulties do *you* experience with providing spiritual care ?”, in the 2003 study (Lundmark 2005, 34).

Category	Subcategory	Sub‐subcategory
One's own approach to spiritual care	Is not himself/herself religious	Difficult to provide something that is not scientifically proved
	Is religious but sees a problem providing spiritual care to patients with a religious affiliation other than one's own	
Feelings of fear or insecurity associated to providing spiritual care	Fear of providing spiritual care	
	Risk of offending the patient or commit abuse	
	Fear of the unknown	
	Fearful of having to open up	
	Feelings of insecurity when faced with spiritual matters	
	Insufficient knowledge	Insufficient education
		Ignorance about spiritual care
		Ignorance about religion and faith
		Ignorance about how to talk about spiritual matters
	Insufficient experience	Lacking experience in providing spiritual care
		Lacking life experience makes it difficult to provide spiritual care
	Insufficient self‐awareness	Must know oneself in order to provide spiritual care
		Must be self‐confident in order to provide spiritual care
		Dare to stand up for one's thoughts and beliefs
Practical difficulties	Lack of resources	
	Lack of peace and quiet	
	Lack of time	
	Lack of space	
	Aggravating routines	
Spirituality does not get space in health care	Spirituality is not prioritised	Greater trust in medicine than in God
		Spirituality is rationalised away, and medicines are given instead
		Patients were not informed about the possibility of receiving spiritual care
	Christians are not provided with sufficient space in healthcare	
	Easy to forget to see the whole person, not just the disease	
Difficult topic to deal with	Difficult topic to talk about	
	Difficult topic to address if the patients do not do it first	
	Spirituality is something very private	
	Difficult to get close enough to the patients	
	It's an art to notice when an individual needs spiritual care	
Not everyone needs spiritual care	Patients don't express spiritual needs	
	Some patients do not want to talk about spiritual problems	
	The need for spiritual care is not what it used to be	

The same working definition of the concept ‘spiritual care’ was used in the 2003 and 2023 studies. The definition was constructed from the results of Strang, Strang et al.'s (2002) study of how Swedish nursing staff at several different healthcare units characterise the spiritual needs of their patients. ‘Spiritual care’ was defined as:

making possible/facilitating for the patient, with the help of suitable nursing interventions, to express and discuss existential questions and to practise his/her spirituality (which may be done through the practising of a specific religion but also through activities which do not need to be of religious nature). (Lundmark 2006, 868).

### 1.1 Societal, Educational and Organisational Changes Relevant to Spirituality and Health Care at the Studied Clinic Since 2003

At a societal level, the spiritual climate in Sweden has changed since 2003. In respect to the World Values Survey (WVS), a comparison of Sweden between ‘Wave 4’ (1999–2003) and ‘Wave 7’ (2017–2022), shows that Sweden in both waves is one of the countries with the highest factor scores for ‘secular values’ and ‘self‐expression’. However, between these two waves, the factor scores for ‘secular values’ increased from 0.9752 to 1.1067 and the factor scores for ‘self‐expression values’ increased from 2.8080 to 3.1133. Between Wave 4 and Wave 7, the proportion of respondents who reported they believe in God decreased from 46.6% to 34.4%. Similarly, the proportion of respondents who reported that they do not believe in God increased from 40.6% to 60.8% (Inglehart et al. 2014; Haerpfer et al. 2022). The distribution of different religious affiliations also changed between the two waves. For example, the number of Muslims in Sweden increased between 2003 and 2023 (Swedish Agency for Support to Faith Communities 2024). To make one international comparison, the population of the United States seemed to be more religious than the population of Sweden during Wave 4. In the World Values Survey 4 (1999–2004), 94.2% of the population of the United States reported to believe in God. In Sweden, on the other hand, the corresponding figure was 46.6% (Inglehart et al. 2014).

At a national level, it is no easy task to establish how and to what extent spirituality and health are addressed in formal healthcare education programs in Sweden and how one might map eventual changes in these programs over time. Notwithstanding this difficulty, at least some observations can be made when one considers various written sources. For example, in 2005, the National Board of Health and Welfare (Swedish: *Socialstyrelsen*) issued a ‘competency description’ for registered nurses. The document described the recommendations made by the Board regarding registered nurses' professional knowledge, competence and approach to their work. These recommendations were used for the planning and development of educational training programs and the preparation of local competency descriptions for registered nurses (Svensk sjuksköterskeförening 2017). One of the many competencies expected of a registered nurse is that they are able to ‘meet the patient's basic and specific nursing needs, physical, psychological, social, cultural, and spiritual’ (author's translation from the original Swedish) (Socialstyrelsen 2005, 11). The National Board of Health and Welfare does not issue such recommendations anymore. Instead, this is done by the Swedish Nurses' Association. In their first recommendation, in 2017, the Association stated that ‘the nurse's competence includes a holistic perspective of the patient's situation, including knowledge of complex needs and problems relating to, for example, communication, cognition, breathing, circulation, eating and nutrition, discharges, personal hygiene, activity and mobility, sleep and rest, pain, and psychosocial, spiritual, and cultural factors’ (author's translation) (Svensk sjuksköterskeförening 2017, 5). In the 2024 revision of these recommendations, *spirituality* is referenced in a similar manner (Svensk sjuksköterskeförening 2024). A corresponding competence description for nursing assistants (nursing auxiliaries with an upper secondary school education in nursing, which today is by far the most common form of nursing auxiliaries in Sweden) was formulated by the National Board of Health and Welfare as late as 2021. This competence description does focus on holistic care, but without mentioning spirituality. Religion is only mentioned in the context of a pronouncement on the importance of respectful treatment, independently of religion or other beliefs (Socialstyrelsen 2021, 9).

Spirituality, religion and spiritual care are not addressed in either the 2003 (commonly named Gy2000) curriculum for the subject ‘Health and social care’ or in the 2023 (commonly named Gy22) curriculum in upper secondary school educations for future nursing assistants (Skolverket 2024). Furthermore, as far as I have been able to establish, spiritual care is not addressed in textbooks used in upper secondary school programs for training nursing assistants in Sweden. This does not mean that spiritual care is never addressed in such educations, but the lack of the concept in the curriculums and textbooks in upper secondary school educations suggests that it is not prioritised.

At the beginning of the millennium, Swedish textbooks used in academic nursing programs (university level) explicitly addressed spiritual care in dedicated chapters, at least in textbooks focussing on palliative care (e.g., Saeteren 2003; Stifoss‐Hansen 2001). *Spiritual care* is also addressed in the introductory textbook commonly used by Swedish education programs for registered nurses from the second and later editions (Lundmark 2014 (and later editions)). However, this has changed over time. In recent academic nursing textbooks that address palliative care, the trend seems to be to replace the concept ‘spirituality’ with the concept ‘existential’ (e.g., Andershed and Ternestedt 2020; Benkel et al. 2024; Österlind et al. 2022). The concept ‘spiritual care’ is not addressed in these textbooks, though Andershed and Ternestedt (2020) do include a chapter on religion in health care in their textbook (Gustafsson 2020).

The trend to replace the concept ‘spiritual’ with the concept ‘existential’ can also be seen when one compares the 2016 and 2024 editions of the Swedish Handbook for Healthcare (an online resource available for all healthcare staff) regarding how palliative care is described. In the 2016 edition of the handbook's section on end‐of‐life care, palliative care is described as ‘alleviating suffering with a holistic view of the patient where physical, psychological, social and existential/spiritual dimensions are included’ (author's translation) (Thulesius 2016). In contrast, in the 2024 edition of the handbook, spirituality is not addressed when palliative care is described: ‘healthcare with the aim of alleviating suffering, and promoting the quality of life, for patients with progressive, incurable disease or injury, and which involves consideration of physical, psychological, social and existential needs, as well as organised support for relatives’ (author's translation) (Janzelius and Yxne 2024).

The oncological clinic that was studied in 2003 and 2023 underwent several organisational changes during the 20 years between the first and second survey. In 2003, besides the radiological and nuclear medicine units, the clinic had three inpatient wards with a total of approximately 65 beds. It also had a small outpatient ward, which was, by far, the smallest unit at the clinic. In 2003, 93 nursing staff (registered nurses and nursing auxiliaries) were on duty at the inpatient and outpatient wards. In 2010, the clinic was subject to a substantial reorganisation, which entailed merging the three inpatient wards into one large ward associated with several units. At the same time, the clinic was moved to newly built premises enabling private rooms for every patient and considerably more space in the common areas. The haematological section was transferred from the internal medicine clinic to the oncological clinic during the same period. The new organisational structure included one inpatient ward with three oncological units, one haematological unit, one oncological outpatient ward and one haematological outpatient ward. Since the haematological unit was not a part of the study in 2003, it was excluded from the 2023 study. With the haematology staff excluded, 82 nursing staff (registered nurses and nursing auxiliaries) were on duty on the inpatient ward and outpatient wards during the 2023 survey. The number of beds on the inpatient ward had decreased to approximately 36 beds. The decrease in inpatient beds was a result of more patients being treated on the outpatient ward.

## 2 The Study

### 2.1 Aims and Research Questions

This article is based on parts of the results of the 2023 replication study of Lundmark's (2003) study and has two aims:

The first one was to replicate the 2003 study with the following research question:

(RQ1) what difficulties do the nursing staff at an oncology clinic perceive with providing spiritual care?

RQ1 provides information on perceived difficulties at the studied oncology clinic in 2023.

The second aim was to perform a comparative analysis of the results from the 2003 data collection and the results from the 2023 data collection to identify similarities and differences between the results, with the research question:

(RQ2) Have the perceived difficulties associated with spiritual care among nursing staff in a holistic nursing context, represented by the oncological clinic that is the object of the study, changed between 2003 and 2023? If so, how?

The answers to these questions are discussed in conjunction with societal changes related to spirituality, changes in the formal education of nurses regarding spirituality and health, and organisational changes at the studied clinic since 2003. Examining these areas will provide us with an indication of whether difficulties associated with spiritual care remain stable over time or whether they are sensitive to the changes mentioned above.

## 3 Methods

The design of the study is a mixed method, longitudinal, descriptive and comparative design, as outlined in the following. Please note that the introduction and descriptions of this study's subjects, method and the discussions thereof follow, in part, the introduction and description of the subjects and method, and discussions in Lundmark (2025).

### 3.1 Procedure

The research procedure of the 2023 study followed the procedure of the 2003 study as closely as possible. However, because the clinic has undergone some organisational changes since 2003 and because of changes in Swedish society during the same period, some adjustments were made to the 2023 study. The change in Swedish society that influenced the design of the 2023 study is the marked increase in the number of Muslims in Sweden between 2003 and 2023, making Islam the second biggest religion in Sweden after Christianity in terms of the number of members of congregations (Swedish Agency for Support to Faith Communities 2024). This change is reflected in how organised religiosity was assessed in the questionnaire, where the words *mosque* and the *Koran* were added next to *church* and the *Bible* as examples of religious institutions and religious texts, respectively. Besides this small change, the questionnaire used in the 2023 study was identical to the 2003 questionnaire.

The questionnaire was distributed as a paper questionnaire, accompanied by a short information letter where an outline of the study was presented, to every registered nurse and nursing auxiliary at the inpatient and outpatient wards (2023: *n* = 82; 2003: *n* = 93) who were on duty during January and February 2003, and January and February 2023. The study participants were also provided with oral information about the study. Participation in the study was voluntary and anonymous. The questionnaires were submitted anonymously by using sealed letterboxes that were located on the wards.

### 3.2 The Questionnaire

The research questions were operationalised to the open‐ended question: What difficulties do you experience with providing spiritual care? (original Swedish: Vilka svårigheter ser DU med att ge andlig omvårdnad?) (cf. Wright 2002). The questionnaire also included an open‐ended question designed to assess how the respondents wished to describe the term spiritual care (Lundmark 2005, 2025), and 17 questions with given alternatives covering some background data and their attitudes towards spiritual care (Lundmark 2006). Some of these 17 questions are accounted for in this article as background data (Tables 2, 3, 4, 5). The questions concerning the background data were inspired by Koenig et al. (2001, 496–500) except the question ‘Do you believe in a life after death?’, which was retrieved from The Fetzer Institute/National Institute on Aging Working Group (1999). Religious belief was assessed by two questions (Table 2). ‘Organised religiousness’ (Table 3) and ‘non‐organised religiousness’ (Table 4) were assessed by one question for each variable. These questions were originally designed before the 2000s, that is before the distinction between religiousness and spirituality was as salient as it has become today (see, e.g., Koenig et al. 2024) and mirror what Schnell (2012) labels as ‘spirituality with religion’ in contrast to ‘spirituality without religion’. Hence, what is assessed here is ‘spirituality with religion’ rather than ‘spirituality without religion’.

**TABLE 2:** Number of years of experience in working in health care. The figures from 2023 are given without parenthesis, and the figures from 2003 are given in parenthesis.

Question	Distribution of responses						
	0–2 years	3–8 years	9–14 years	15–20 years	More than 20 years	Missing answers	*n*
How many years have you worked in health care?	6% (7%)	11% (19%)	17% (12%)	17% (22%)	49% (40%)	0 (0)	47 (68)

**TABLE 3:** The respondents' reported belief in God and belief in life after death. The figures from 2023 are given without parenthesis, and the figures from 2003 are given in parenthesis. The proportion of staff members who reported that they believed in God decreased between 2003 and 2023 by a statistically significant amount (*p* = 0.034, Fisher's exact test) at *p* = 0.05. The decrease of belief in life after death are not significant (*p* = 0.10, Fishers exact test) at *p* < 0.05.

Questions	Distribution of answers				
	Yes	No	Do not know	Missing answers	*n*
Do you believe in God?	40% (53%)	47% (24%)	13% (22%)	0 (1)	47 (67)
Do you believe in life after death?	34% (56%)	30% (22%)	36% (22%)	0 (0)	47 (68)

*Note:* In the test, the columns Yes and No were tested.

**TABLE 4:** Organised religiousness of the respondents. The figures from 2023 are given without parenthesis, and the figures from 2003 are given in parenthesis. The differences between 2003 and 2023 are not statistically significant (*p* = 1.0, Fisher's exact test) at *p* < 0.05.

Question	Distribution of answers						
	Never	At most once a year	A few times per year	Around once or twice a month	About every week	Missing answers	*n*
How often do you attend religious meetings (e.g., in a church or mosque)?	30% (22%)	45% (37%)	11% (27%)	6% (6%)	8% (7%)	0 (1)	47 (67)

*Note:* In the 2003 study, the question was formulated as: “How often do you go to church or attend religious meetings?” In the cross‐tabular analysis related to Fisher's exact test, the alternatives “a few times per year”, “at most once a year” and “never” have been combined as well as the alternatives “around once or twice a month” and “about every week”.

The questionnaire was validated during the 2003 study within the scientific community through discussions with experienced researchers in nursing science, the social science of religion and statistics (cf. Kvale 1997). The questionnaire was tested in a small pilot study, where six caregivers were asked to complete it and provide feedback on its structure and layout, which in turn was used to increase the clarity of the questionnaire. The questionnaire (in Swedish) can be found in Lundmark (2003).

### 3.3 Analysis

The subsection of the questionnaire responses presented in this article was analysed using content analysis as described by Downe‐Wamboldt (1992). The answers to each question were compiled and printed out. The responses were then read through to identify general patterns in the responses. Next, the answers were reread and divided into meaningful units based on parts of sentences, whole sentences or even several sentences that substantively formed a unit. These substantive units were then condensed and grouped into subcategories (in the case of the 2003 study: into sub‐subcategories, then subcategories) which, in turn, were grouped into categories. The questionnaire answers were then read once again and compared with the categorisation schema in order to verify that the content analysis covered every aspect of the participants' responses (Downe‐Wamboldt 1992; Lundmark 2005). The procedure in both 2003 and 2023 was carried out by one (and the same) researcher with profound knowledge about the topic in accordance with Tuval–Mashiach's recommendations concerning a research material, which is coded by one researcher only (Lieblich et al. 1998, 133). The 2023 results were compared with the 2003 results. The Statistical Package for the Social Sciences (2023: SPSS 29.0.1.0 and 2003: SPSS 11.00) was used, including Fisher's exact test to generate descriptive statistics.

### 3.4 Ethical Considerations

The study was approved by an ethics committee (Etikprövningsmyndigheten, Dnr 2022‐05805‐01). The head of the clinic and the operation managers for the wards also gave their approval for the study. Participation in the study was voluntary and anonymous.

### 3.5 Reflexivity

As the sole researcher in the presented parts of the 2023 replication study, the research process is perhaps even more sensitive to my own beliefs, background and experiences. Those being deemed as relevant to account for are the following: I am myself a practicing religious person and I have worked 28 years as a registered nurse (mostly night shifts) at the same oncology clinic where the study is conducted. I stopped working at the clinic the year before the replication study took place. Throughout most of these years, I worked part‐time at the clinic and part‐time as a PhD student (Psychology of Religion). Later, I combined part‐time work as a registered nurse with a tenured associate professorship in Religious Studies. The research interest leading up to the presented study comes from my ‘embodiment’ of the academic fields of Religious Studies and Nursing, and the lived experiences of the same.

## 4 Results

### 4.1 Response Rate

In the 2003 study, the response rate was 73% (68 out of 93 staff). However, the response rate varied between the four oncology wards that participated in the study (94%, 80%, 60% and 59%, respectively). Of the staff who handed in a questionnaire, a total of 46 (68%) answered the question regarding perceived difficulties in providing spiritual care. In the 2023 study, the overall response rate was 57% (47 of 82 staff). However, the response rate also varied between the different wards. For the inpatient ward, the response rate was 45% (22 of 49 staff), while the response rate for the outpatient ward was 76% (25 of 33 staff). Of the participating staff who handed in the questionnaire in the 2023 study, 34 (72%) answered the question regarding perceived challenges in providing spiritual care.

### 4.2 Subjects

Among the 47 respondents in the 2023 study (compared to 68 in the 2003 study), 73% (34 respondents) were registered nurses (up from 57% in 2003), while 27% (13 respondents) were nursing auxiliaries (down from 40% in 2003). Table 2 presents how many years the respondents had worked in health care. In respect to this variable, the distribution was quite similar in 2023 to what it was in 2003. According to one of the operation managers, approximately one‐third of the staff who were working in 2023 had worked at the clinic for at least 20 years and, thus, could have also participated in the 2003 study.

The variable ‘religious belief’ was measured by two questions: *Do you believe in God? (yes/no/do not know)* and *Do you believe in life after death?* (*yes/no/do not know*). The distribution of the answers to these questions is presented in Table 3. The proportion of staff members who reported that they believed in God decreased between 2003 and 2023 by a statistically significant amount (*p* = 0.034). The change in the distribution of the responses concerning belief in life after death was echoed by the responses regarding a belief in God between 2003 and 2023. However, these changes are not statistically significant. The distributions of the variables ‘organised religiousness’ and ‘non‐organised religiousness’ can be seen in Tables 4 and 5. Regarding organised religiousness, the staff were less engaged in the asked for religious activities in 2023 compared with 2003. Concerning the variable ‘non‐organised religiousness’, the differences in distribution between 2003 and 2023 were less pronounced. These differences are not statistically significant.

**TABLE 5:** Non‐religiousness of the respondents. The figures from 2023 are given without parenthesis, and the figures from 2003 are given in parenthesis. The differences between 2003 and 2023 are not statistically significant (*p* = 0.42, Fisher's exact test) at *p* < 0.05.

Question	Distribution of answers						
	Never	A few times per year	A few times a month	Some times a week	Every day	Missing answers	*n*
How often do you spend time with activities like praying, meditating, or reading religious literature (e.g., the Bible or the Koran)?	53% (47%)	19% (18%)	9% (6%)	13% (19%)	6% (10%)	0 (1)	47 (67)

*Note:* In the 2003 study, the question was formulated as: “How often do you spend time with activities like praying, meditating, or reading the Bible?” In the cross‐tabular analysis related to Fisher's exact test, the alternatives “a few times per year” and “never” have been combined as well as the alternatives “a few times a month”, “sometimes a week” and “every day”.

Concerning opinions and attitudes towards spiritual care, the results between 2023 and 2003 were similar, displaying only minor variations in the respondents' opinions about holistic care. To a high degree, the respondents considered that holistic care is important in nursing and that such holistic care should include spirituality. However, fewer respondents felt holistic care was provided on their ward in 2023 compared to 2003. The respondents' opinions on whether nursing staff should engage in providing spiritual care increased from approximately two‐thirds of the staff in 2003 to three‐quarters in 2023. The respondents' estimations of whether the nursing staff on their ward provided spiritual care were similar in 2003 and in 2023.

Change was more apparent regarding self‐reporting of how often the respondents themselves provided spiritual care. Those who claimed to provide spiritual care ‘often’ or even ‘daily’ increased from approximately 3% in 2003 to 10% in 2023. Respondents who ‘never’ or ‘hardly ever’ performed spiritual care decreased from approximately 54% in 2003 to approximately 40% in 2023. Across the same period, the respondents' self‐reported ability to provide spiritual care only slightly increased in 2023 compared to 2003. Education in spiritual care is considered ‘quite important’ in 2003 and 2023, with a slight increase in importance recorded in 2023.

In contrast to this, a smaller number of respondents in 2023 reported that they had received education in spiritual care, either during their formal education or while performing their work. Concerning the experience of ‘being at ease when providing spiritual care’ or ‘being not at ease when providing spiritual care’, no significant differences between 2003 and 2023 were reported. (Lundmark 2025).

### 4.3 A Comparison of the Results of the Nursing Staff's Perceived Difficulties Associated With Spiritual Care in the 2003 Study and the 2023 Study

The content analysis of the respondents' answers to the question ‘What difficulties do you experience with providing spiritual care?’ generated six main categories in the 2003 study (Table 1) and four main categories in the 2023 study (Table 6). In the following section, a comparison of these results will be conducted, with a starting point in the 2023 categorisation. A discussion of the results of the 2003 study can be found in Lundmark (2003, 2005).

**TABLE 6:** Categories and subcategories generated from the content analysis of the answers to the question: “What difficulties do *you* experience with providing spiritual care ?” in the 2023 study. (In contrast to the 2003 study, the results are presented as categories and subcategories only.)

Category	Subcategory
Practical or structural difficulties	Lack of time Healthcare environment, limited physical space for one‐on‐one conversations Stress: (i) not enough time, and (ii) patients observe the stress of the healthcare staff and thus refrain from addressing spiritual needs Staff shortages Advanced medical treatment is provided at the cost of psychosocial and spiritual perspectives Too many (staff) involved in the healthcare process Challenges in communicating between different occupational categories Lack of formal education and support for the healthcare staff Other patient needs have a higher priority
Lack of knowledge and education	Knowledge and education, knowing how to act regarding questions about spiritual and/or existential needs Unclear whose job it is Lacking the right competence Difficulties in interpreting or understanding patients' individual needs Difficult to guide the patients to the appropriate authorities or individuals Difficult to initiate conversations about spirituality Difficult to see the whole person
One's own approach can have a negative impact	Fear of offending or abusing the patient and insecurity about when it is right or wrong to talk about spiritual matters Difficult because of the dependence of one's own openness in order to understand and dare to approach spiritual questions Fear among the staff Unknown “concept”, can't understand “such questions” when oneself does not believe in religion or spirituality Uninterested staff
Society's perceived attitude can have negative impact	Society is built on neutrality. Then you have to be very careful to mention spiritual care

#### 4.3.1 Practical or Structural Difficulties

This category was the largest, accounting for approximately half of the statements made by the respondents. The most common statements concerned a lack of time (to address spiritual care), followed by stress. Stress, as a problem related to spiritual care, was reported on in two different ways: either as an aggravating circumstance, when the staff member wanted to provide spiritual care, or as a circumstance where the staff member noticed that patients refrained from addressing existential questions when they saw that the nursing staff was under stress. Other subcategories include remarks about the physical environment, especially concerning the lack of suitable locations for private conversations. There are also statements about other structural difficulties, for example, in relation to communication between different professions.

Some respondents mentioned that too many different people were involved in the care of the patients, while others claimed that there was a lack of staff on the wards. The observation was also made that advanced medical treatments are sometimes administered at the cost of psychosocial and spiritual perspectives. Furthermore, some respondents argued that other (unspecified) patient needs were prioritised higher than the patients' spiritual needs. In this context, a lack of staff supervision was also mentioned.

This category has its equivalent in the 2003 study with the category ‘practical difficulties’, and with the subcategory ‘spirituality is not prioritised’, subsumed under the category ‘spirituality does not get space in health care’. In the 2003 study, the category ‘practical difficulties’ was also the largest category. Note that ‘stress’ was not mentioned in the 2003 study.

#### 4.3.2 Lack of Knowledge and Education

This category accounts for approximately one‐fourth of the statements in the 2023 study. The largest subcategory within this category includes ‘knowledge and education’ and the difficulties the staff face in knowing how to act when they are confronted with their patients' existential needs. Another subcategory addresses issues regarding ‘competence’. ‘Competence’ was kept distinct from ‘knowledge’ as a subcategory because ‘competence’ is understood as being broader than ‘knowledge’. For example, it is possible to understand ‘spiritual competence’ both as a form of knowledge and a social skill. In this category, we also find such perceived difficulties as ‘difficulties in interpreting or understanding their patients’ individual needs'; ‘challenges in directing patients to the appropriate service based on their specific needs’; ‘a lack of ability to be open to engage in this type of conversation’, and ‘a lack of ability to perceive the patient as a whole person’.

In the 2003 study, several statements addressed this same theme, including ‘a lack of knowledge’ and ‘a lack of experience’, which, in the 2003 study, were categorised under the main category ‘feelings of fear or insecurity associated with providing spiritual care’. In the 2023 study, there are more of these kinds of statements, and in the analysis, another assessment concerning the special position of the category is made vis‐à‐vis, for example, feelings of fear or insecurity (see the next category). To a large extent, the 2003 category ‘difficult topic to deal with’ overlaps with the 2023 category ‘lack of knowledge and education’.

#### 4.3.3 One's Own Approach Can Have Negative Impact

This category also accounts for approximately 1/4 of the respondents' statements. Subsumed by this category are subcategories that address fear; for example, ‘fear of offending the patient’ and ‘difficulty in knowing when it is appropriate to address spirituality’. The category also includes ‘the experience of religion and spirituality as an “unknown” concept’ for some of the staff. Statements concerning the influence of the nursing staff being uninterested in spiritual matters were also noted. Several statements were made concerning openness to spiritual matters as an essential condition for understanding and daring to approach spiritual questions.

A similar category was also found in the 2003 study. However, statements about ‘feelings of fear or insecurity associated with providing spiritual care’ (which were grouped into their own category in 2003) were categorised in 2023 as a subcategory of this category.

#### 4.3.4 Society's Perceived Attitude Can Have Negative Impact

Even though this category contained only one statement, giving it its own category is still justified because the statement was entirely distinct from all the others. The category statement was formulated by the respondent as: ‘Society is built on neutrality. Because of this, you have to be very careful when mentioning spiritual care. This has become very clear during the last 10–15 years’. This statement is interpreted as expressing the idea that society's attitude limits the possibility of providing spiritual care.

There was no equivalent to this category in the 2003 study. In the 2023 study, there was no equivalent to the final category listed in the 2003 study, namely, ‘not everyone has a need for spiritual care’.

## 5 Discussion

### 5.1 On the Results

The similarities between the 2003 and 2023 studies are striking. The differences between the studies are primarily due to some difficulties being more pronounced in 2003 and others in 2023. This accounts for the categorisations in 2003 and 2023 manifesting in somewhat different ways despite their underlying similarities.

This observation is only partly in agreement with other parts of the replication study, reported elsewhere, where it was found that attitudes towards spiritual care and the understanding of the concept of ‘spiritual care’ had Mötet med Religion i det Nya Sverige (in Swedish) [The Encounter With Religion in the New Sweden] had changed in subtle yet distinct ways since 2003 (Lundmark 2025). That perceived difficulties associated with spiritual care did not substantially change is somewhat surprising considering the societal changes in Sweden that occurred during the 20 years between the studies and the organisational changes that were implemented at the clinic during the same time. The most noticeable difference among the staff between 2003 and 2023 was the decrease of spirituality (which here should be regarded primarily as spirituality with religion) which followed the general trend of increasing secularisation in Sweden, even though the number of staff who reported to believe in God or life after death and reported that they regularly participated in religious activities or participated in religious meetings was still higher at the oncological clinic than the Swedish national average. Changes in the staff members' spirituality were thus not reflected in changes in what were perceived as difficulties associated with spiritual care.

‘Lack of time’ was the most common difficulty mentioned in both the 2003 and 2023 studies and is also in agreement with previous research on this topic, where ‘time’ was identified as a highly influencing factor for whether spiritual care is provided or not (Mascio et al. 2022; Balboni et al. 2014). ‘Stress’, on the other hand, was not mentioned in the 2003 study, whereas it was mentioned several times in the 2023 study. However, it is not unreasonable to argue that a lack of time can generate stress, and thus, stress could have been a problem in 2003, even if it had not been explicitly identified as such. Another possible interpretation is that the lower level of spirituality reported by the 2023 staff had a negative impact on stress management on a group level (i.e., more of the staff experience stress) (cf. De Diego‐Cordero et al. 2022).

A lack of suitable locations where staff members could engage in private conversations with patients was mentioned in the 2003 study and the 2023 study as an aggravating circumstance. This observation is in agreement with previous research, for example, where a ‘lack of private space’ was identified as a barrier to providing spiritual care (Balboni et al. 2014). The organisational changes that have taken place at the clinic have not obviated the identified difficulties, despite more space and an increased number of staff in relation to numbers of patients.

Another challenge to the provision of spiritual care at the clinic, which has also been highlighted in previous international research (Mascio et al. 2022; Jones et al. 2021; Balboni et al. 2014), and was more pronounced in 2023 than in 2003, was the perceived lack of knowledge and education regarding spiritual care. This state of affairs existed despite the fact that, since 2014, the topic of spirituality in health care and spiritual care is addressed in a chapter on spirituality in the introductory textbook commonly used in Swedish registered nursing educational programs (Lundmark 2014 (and later editions)), as well as in several textbooks on palliative care that were published at the beginning of the millennium. The trend in recent textbooks on palliative care has been to replace the concept ‘spirituality’ with the concept ‘existential’. However, this change can hardly, in such a short time, have made any significant impact on a lack of knowledge about spiritual care, though it remains a concern for the future. Regarding nursing assistants (in the 2023 material the only existing form of nursing auxiliaries represented in the study), it is hard to establish if and to what extent spiritual care is addressed in upper secondary school programs for training nursing assistants in Sweden. However, one can notice the lack of the concept ‘spirituality’ and ‘spiritual care’ in the competence descriptions of nursing assistants, in the curriculum for the subject ‘Health and social care’ in upper secondary school educations for training nursing assistants and, as far as I have been able to determine, a lack of the same in the various textbooks used in upper secondary school programs for training nursing assistants.

It seems reasonable to conclude that there is still much to be done to obviate difficulties associated with spiritual care, not only concerning practical and structural difficulties but also concerning the perceived lack of knowledge and education.

### 5.2 On Methods, Limitations, Strengths, Generalisability and Future Research

The low response rate in 2023 to the questionnaire at the clinic's inpatient ward can be attributed to the condition that the participants filled in the questionnaire while they were at work. The workload on the inpatient ward at the time of the study was higher than that on the outpatient ward, according to the operation managers. However, it is also possible that a potential bias emerged since we do not know whether those participants who filled in the questionnaire represented those nursing staff who held the strongest opinions about spiritual care (either against, or for, spiritual care). In Section 3.5, when addressing matters of reflexivity, I mentioned that I have myself worked for a long period at the oncology clinic in question until the year prior to the replication study. Though the advantages of having the kind of insider perspective that I still had when the study was conducted are obvious (e.g., having access to the research field in multiple ways), there are also disadvantages. For instance, the researcher knows some of the study's potential participants. This circumstance could potentially create a feeling of pressure to participate in the study notwithstanding it being anonymous. However, the relatively low response rate can be interpreted as the potential participants' feeling that they were not obliged to participate, even if they did know the researcher. As far as I can judge, my close connection to the studied clinic has not created any biases either in terms of methods, results or discussions, other than that the study would never have been conducted (at least not by me), if I didn't have this close connection to the clinic, including an understanding of what it means to work with oncology patients and the spiritual dimensions of that work.

As noted in the Method section, the distinction between ‘spirituality’ and ‘religiousness’ was not as distinct at the time of the 2003 study as at the time of the 2023 study. This becomes apparent in how the background information on the participants' own spirituality was assessed in the 2003 study, where Lundmark primarily addressed what Schnell (2012), calls ‘spirituality with religion’, while omitting ‘spirituality without religion’. If the 2023 study only focused on how the nursing staff perceived the difficulties providing spiritual care in 2023, it would, admittedly, have added valuable information to have included background questions in the questionnaire that also assessed spirituality without religion. However, since the focus of the 2023 study was on changes over time in respect to perceived difficulties associated with spiritual care, being as faithful as possible to the original study's design was deemed to be of primary importance. This methodological choice may have come at the price of a decreased amount of background information concerning each informant's stance concerning ‘spirituality without religion’. Notwithstanding this, there is sufficient information to compare the oncology clinic during the 2003–2023 time period with the relevant waves of the WVS because, when assessing secular values, the WVS also asked for what, following Schnell's terminology, would be labelled ‘spirituality with religion’.

While the present study has identified several difficulties associated with spiritual care at a specific clinic, the question remains whether we can also learn something about these difficulties that is valid beyond the confines of this particular clinic. Given the nature of this study (a longitudinal case study following one specific oncology clinic), the question of generalisability can be reformulated as a question concerning transferability, that is, can the reader transfer knowledge generated from the given case to another similar case? (Mertens 2005). One criterion for a study's validity in the context of qualitative research is to ensure that the study has sufficient depth and breadth to generate additional insights into the issue that is being researched (Yardley 2015). In the present study, depth has been ensured by providing (i) rich background information about the clinic; (ii) some relevant information from the World Values Survey about Swedish society; (iii) information on changes in how spirituality is presented in various written sources in relation to health care in a Swedish nursing context; (iv) information about the study's theoretical framework and method; and (v) a detailed account of the results of the study. Consequently, the reader has been provided with sufficient information to make their own comparisons with other cases (Mertens 2005). Regarding this, two issues should be considered in particular. First, it is essential to consider Sweden's extreme ranking in the WVS. The extent to which the results of the present study can, in a meaningful way, be transferred to other cases in other countries should be informed by the similarities and differences between Sweden and the other countries in question. Second, it is vital to consider the presence of palliative elements at oncology clinics since such palliative elements might function as a catalyst for reflections on spirituality and spiritual health, making these reflections more pronounced in an oncological context compared to healthcare contexts where palliative care is not as common (Lundmark 2006, 2025; Mascio et al. 2024).

In this study, the question of changes in the nurses' formal education regarding spirituality and health care has only been mirrored against formal documents and the content in textbooks. To get a fuller understanding of how education in spiritual care is implemented over time in nursing education programmes (both in upper secondary school education and in university studies), the content in formal documents and in textbooks needs to be complemented by, for example, interviews and/or surveys (cf. Jones et al. 2021). This is a recommendation for further studies.

To the best of my knowledge, the present study is the first longitudinal study of changes in respect to nursing staff's perceptions of difficulties associated with spiritual care; hence, the lack of studies with which we can compare results. This raises a question: would the results be similar if the question were investigated longitudinally in less secularised areas of the world, or in nursing contexts where formal education in spiritual care has a more prominent role than in the Swedish case?

## 6 Conclusions

The difficulties associated with spiritual care at the oncology clinic studied here remained largely unchanged between 2003 and 2023. This suggests that these difficulties might not be significantly influenced by the societal shifts in spirituality that occurred over the past 20 years in Sweden; in particular, the rise in secularisation. This observation suggests that the nurses' spirituality might not be a reliable predictor for perceived difficulties associated with spiritual care. The organisational changes that were implemented at the clinic might not be a reliable predictor for perceived difficulties either. Furthermore, the small changes in how spirituality is presented in relation to health care in the Swedish nursing context, for example in nursing textbooks and official documents, also do not seem to play a significant role in this context. The identified difficulties can be summarised as (i) practical or structural difficulties, including a lack of time, experiences of stress, a lack of suitable locations for private conversations, and difficulties in communicating between different professions; (ii) a lack of knowledge and education; and (iii) one's own approach to spirituality being perceived as having a negative impact.

Much work to obviate the difficulties associated with spiritual care still needs to be done. The findings of this study are relevant when considering suitable actions in this regard.

## Conflicts of Interest

The author declares no conflicts of interest.

## Data Availability

The data that support the findings of this study are available from the corresponding author upon reasonable request.
